# Influence of Bovine Serum Albumin-Flavonoid Interaction on the Antioxidant Activity of Dietary Flavonoids: New Evidence from Electrochemical Quantification

**DOI:** 10.3390/molecules24010070

**Published:** 2018-12-25

**Authors:** Rui Geng, Lei Ma, Liangliang Liu, Yixi Xie

**Affiliations:** 1 Institute of Bast Fiber Crops, Chinese Academy of Agricultural Sciences, Changsha 410205, China; 201610141365@smail.xtu.edu.cn; 2 Key Laboratory for Green Organic Synthesis and Application of Hunan Province, College of Chemistry, Xiangtan University, Xiangtan 411105, China; 3 Zhengzhou Research Base, State Key Laboratory of Cotton Biology, Zhengzhou University, Zhengzhou 450001, China; malei@caas.cn

**Keywords:** antioxidant capacity, BSA-flavonoid interaction, CRAC assay, DPPH assay, FRAP assay

## Abstract

Interaction between dietary flavonoids and albumins plays an important role in the bioavailability and bioactivity of flavonoids. Therefore, the influence of this interaction on the antioxidant activity of flavonoid has attracted much interest. In this study, a ceric reducing/antioxidant capacity assay (CRAC) was employed to investigate the effects of albumin-flavonoid interaction on the antioxidant activity of seven common flavonoids. The results obtained from the CRAC assay were also compared separately with the results from the spectrophotometric methods including 2,2-diphenyl-1-picrylhydrazyl (DPPH) and ferric reducing antioxidant power (FRAP) assays. All the flavonoids show a decreasing in the antioxidant activity detected by CRAC assay, indicting a “masking effect” of bovine serum albumin (BSA)-flavonoid interaction. However, the results from DPPH and FRAP assays were conflicting, which may be attributed to the influence of solvent systems.

## 1. Introduction

Flavonoids are the major class of polyphenols with a chemical structure based on a common skeleton of phenyl-benzo-γ-pyran (C_6_-C_3_-C_6_), also named flavan nucleus, containing of two phenyl rings (A and B) connected through a pyran ring (C). Thus far, more than 8000 kinds of flavonoids have been isolated from various plants, and many of them are responsible for the colors of flower, fruits and leaves [1]. Flavonoids are daily ingested by human from vegetables, fruits, beverages and other plant-derived foods. Flavonoids are well known as natural antioxidants, and epidemiological studies have indicated that a diet rich in flavonoids may reduce the incidence of cardiovascular and cerebrovascular diseases, and may also attenuate the progression of diabetes or aging [2]. The bioactivities of flavonoids are highly depended on their bioavailability and bio-accessibility, which are closely related to the absorption in the intestine and the interactions with proteins in the blood [3,4].

Serum albumin is the most abundant carrier protein in the blood. Flavonoids entering into the blood will mostly bind with serum albumin before being transported to the organs and tissues [5,6]. The interactions between serum albumin and flavonoids are expected to modulate the bio-availability of flavonoids and also influence their bioactivities, especially the antioxidant capacity [7,8]. Hence, the influence of the interaction between albumin and flavonoids on the antioxidant activities of flavonoids has attracted great interest among researchers [9,10]. Arts et al. investigated the antioxidant activities of three flavonoids in the plasma with the trolox equivalent antioxidant capacity assay and found a masking of antioxidant activity by the protein-flavonoid interaction [11]. Another study by Cao et al. illustrated that plasma proteins masked most of the investigated dietary polyphenols, thus reducing their radical scavenging potential; however, the results among the four employed antioxidant assays were quite different [12]. Zou reviewed the influence of polyphenol-plasma protein interaction (PpPI) on the antioxidant activity of polyphenols, and pointed out that the influence of PpPI on the antioxidant activity of polyphenols showed different trends which were decided by both the kinds of antioxidant assay and polyphenols [13]. In fact, the results obtained from different antioxidant assays were hardly comparable because of the different mechanisms, pH and solvent [14]. Moreover, the color of flavonoid and the turbidity caused by protein in some solvent systems may bring big problems to the determination accuracy of the antioxidant capacity, especially for the antioxidant assays based on spectrophotometric methods [15]. 

As alternatives to traditional spectrophotometric methods, electrochemical methods have been used to determine the antioxidant capacity of flavonoids in recent years. Most of the usual electrochemical techniques have been employed to detect antioxidant capacity, including cyclic voltammetry (CV), square wave voltammetry (SWV), differential pulse voltammetry (DPV) and chronoamperometry (CA) [16]. Among them, CA is a very simple and effective way to evaluate the antioxidant capacity by measuring the decline of oxidant concentration in the system [17]. Ferreira et al., investigated the antioxidant activities of five free flavonoids and their Fe^2+^ complexes with a so-called ceric reducing/antioxidant capacity assay (CRAC), which measures the consumption of the strong oxidants Ce^4+^ by CA for directly quantifying the reducing power of the antioxidant samples [18]. The CRAC method has also been used to study the structure/antioxidant activity relationship of eight flavonoids, and the results are highly consistent with to the quantitative structure-property relationships [19]. These results indicate that the CRAC assay is an effective and suitable method to evaluate the antioxidant capacity of flavonoids.

Electrochemical methods including CV and CRAC assay were employed to study the influence of protein-flavonoid interaction on the antioxidant capacity. The antioxidant activities of seven common dietary flavonoids and a contrast trolox were measured and bovine serum albumin (BSA) was used as model protein. The results obtained from CRAC assay were also compared separately with the results from the spectrophotometric methods including 2,2-diphenyl-1-picrylhydrazyl (DPPH) and ferric reducing antioxidant power (FRAP) assays. 

## 2. Results and Discussion

### 2.1. CV Assay

**Figure 1:**
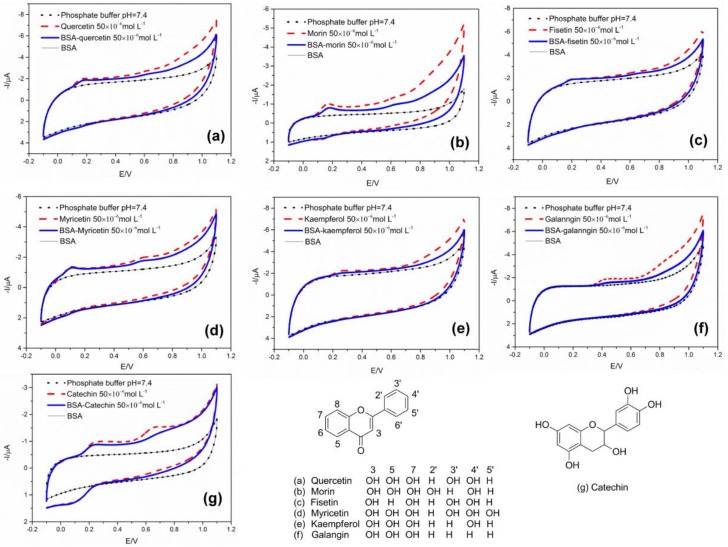
The structures of investigated flavonoids and their cyclic voltammetries (CVs) before (red line) and after (blue line) binding to bovine serum albumin (BSA), (**a**): quercetin, (**b**): morin, (**c**): fisetin, (**d**): myricetin, (**e**): kaempferol, (**f**): galangin, (**g**): catechin.

CV can reflect the redox reaction tendency of flavonoids which correlated to their antioxidant activities. The flavonoids with lower oxidation potentials more easily donate electron(s), and therefore, are regarded to be more potent antioxidants [20]. Figure 1 shows the structures of the studied flavonoids and their CVs before (red line) and after (blue line) binding to BSA, respectively. As seen, all the flavonoids except kaempferol have two oxidation peaks ranging from 0.1 V to 0.8 V. Take the first oxidation peak in compassion, it ranks as follows: myricetin (0.16 V), quercetin (0.18 V), morin (0.19 V), fisetin (0.20 V), catechin (0.25 V), kaempferol (0.28 V) and galangin (0.45 V). This result is generally in agreement with the literature data shows a rank of that myricetin (0.30 V), quercetin (0.39 V), fisetin (0.39 V), kaempferol (0.44 V), catechin (0.45 V) and galangin (0.59 V) [21]. The CV curve of BSA alone is almost consistent with the bottom fluid curve (black line), which means BSA was not oxidized or reduced during the CV scan and indicates that BSA did not interfere with the electronic signal of flavonoids.

When comparing the anodic waves of flavonoids with those of the corresponding BSA binding flavonoids, almost all the flavonoids show a decreased oxidation peak height in different degrees, especially morin, galanngin and catechin. This means binding to BSA minimized the chances of flavonoids to be oxidized; BSA had a certain masking effect for flavonoids [11]. Moreover, since the different oxidation peaks are related to hydroxyl groups at different positions, the CV curves may indicate the possible binding sites of flavonoids to BSA. For example, kaempferol has an oxidation peak at 0.28 V, which is related to the oxidation of hydroxyl group at 4’ position, while morin has two oxidation peaks at 0.19 V and 0.6 V, which are, respectively, related to the oxidation of hydroxyl groups at 4’ and 2’ positions [22]. As seen in the CVs of kaempferol and morin, the oxidation peak of kaempferol only slightly decreased after binding to BSA, while both the two oxidation peaks of morin apparently decreased after binding to BSA, and the second peak decreased more than the first one. These results indicate that morin may have higher binding affinity for BSA than kaempferol, and hydroxyl group at 2’ position may play more important role in binding to BSA.

### 2.2. CRAC Assay

**Figure 2:**
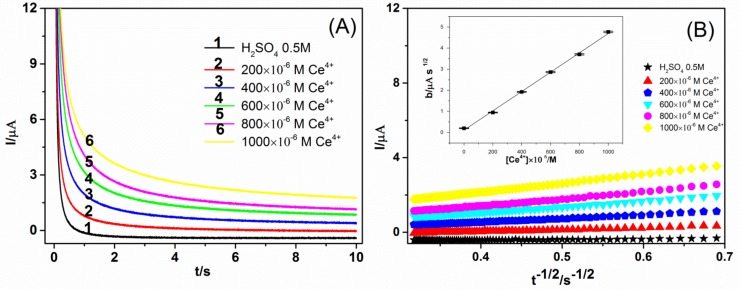
(**A**) Current–time response signal (chronoamperograms) recorded for the supporting electrolyte (H_2_SO_4_ 0.5 mol L^−1^) and for Ce^4+^ solutions at different concentrations. (**B**) Data plotted as *I* versus *t^−1/2^* (Cottrell lines) recorded for the supporting electrolyte (H_2_SO_4_ 0.5 mol L^−1^) and for Ce^4+^ solutions at different concentrations. Inset with values of the slopes *b* for the Cottrell lines plotted against Ce^4+^ concentrations.

Figure 2A shows the results of chronoamperometric measurement with the Ce^4+^ solutions at different concentrations in 0.5 mol L^−1^ H_2_SO_4_.The height of current curve is positively related to the concentration of Ce^4+^. Figure 2B is the corresponding Cottrell lines, and the insert is the values of the slopes against Ce^4+^ concentration. According to regression analysis of the values displayed in the Figure 2B, a mathematical expression was obtained:

(1) $$b=0.11\mu As^{1/2}+4.58\times 10^{3}\mu As^{1/2}/M\times [Ce^{4+}]M$$

where *b* is the Cottrell slope of the experimental value. [Ce^4+^] is the concentrations of Ce^4+^ in solution. Then, the CRAC value can be obtained by the equation (2) and can be presented by Trolox equivalent (*TE*):

(2) $$CRAC_{value}=1\times 10^{3}-\frac{b-0.11}{4.58\times 10^{3}}$$

(3) $$TE=\frac{CRAC\;Value_{flavonoid}}{CRAC\;Value_{Trolox}}$$

**Figure 3:**
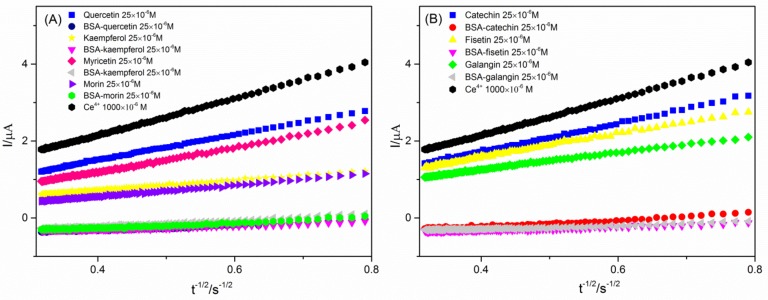
Dependence of *I* on *t^−1/2^* based on the Cottrell equation used to determine *b* for the oxidizing solution, before and after BSA-flavonoid complex formation, (**A**) for quercetin, kaempferol, myricetin and morin, (**B**) for fisetin, galangin and catechin.

Figure 3 illustrates the dependence of *I* on *t^−1/2^* for the most representative results that was used to produce the value of *b* in the oxidizing solution before and after BSA-flavonoid complex formation. According to the curve, we can obtain the antioxidant capacity of the flavonoids before and after formation of a complex with BSA, and obtain the affinity between different flavonoids and BSA. The Trolox equivalent (TE) values represent the antioxidant activity of flavonoids. The Cottrell slopes of the flavonoids, the CRAC values and the TE values obtained from Figure 3 were all presented in the Table 1. The TE values of flavonoid after bound with BSA in the table are subtracted from the background BSA. As seen, the antioxidant capacity of the free flavonoids is ranked as: kaempferol > morin > galangin > fisetin > myricetin > quercetin > catechin, while after binding with BSA, it ranks as: kaempferol > fisetin = galangin > quercetin > morin > myricetin > catechin.

### 2.3. Comparison of CRAC Assay and Spectrophotometric Assay

In order to further study the masking effect of BSA on flavonoids, two traditional antioxidant activity evaluation methods including DPPH assay and FRAP assay were performed. The results are showed in Table 2. All the studied flavonoids showed certain reducing power in FRAP assays. In DPPH assay, morin and galangin were too weak in scavenging the DPPH free radical to obtain their IC_50_ values. For comparing the antioxidant activity changes, FRAP values of flavonoids were normalized as TE values from dividing them by trolox FRAP value. Similarly, the IC_50 _values of flavonoid from DPPH assay were also normalized as Trolox equivalent values (1 mol flavonoid equals to *TE* value mol trolox in scavenging DPPH free radicals) by dividing IC_50_ value of trolox by them. Table 3 shows the changes of the TE value (ΔT) of different flavonoids after binding with BSA. The positive values indicate an increasing in the antioxidant capacity, whereas the negative values denote a decreasing in that. For the results of DPPH assay, only myricetin and kaempferol showed a masking effect, and the others either could not be calculated or show an enhancing effect. These results are in agreement with the conclusions drawn by Cao et al. in their review that the results of DPPH assay are sometimes conflicting [23]. Similar contradictory results were also seen in FRAP assay, which all the flavonoids except for quercetin showed an “enhancing effect” in their antioxidant activity.

For the CRAC results, all the flavonoids showed a decreasing in the antioxidant activity, indicting a masking effect of BSA-flavonoid interaction. Among them, the CRAC values of morin, galangin and catechin decreased by 82.2%, 67.6% and 67.0%, respectively, which is in agreement with their significant decreases of the oxidation peaks in CV. The CRAC values of myricetin and kaempferol also decreased by approximately 70.1% and 76.0%, while their oxidation peaks in CV did not decrease significantly, indicating that the CRAC method were more effective to detect the masking effect of BSA on flavonoids.

## 3. Materials and Methods 

### 3.1. Chemicals and Reagents

Catechin, quercetin, myricetin, fisetin, galangin, kaempferol, morin, trolox, bovine serum albumin (BSA), 2,2-diphenyl-1-picrylhydrazyl (DPPH) and 2,4,6-tri(2-pyridyl)-1,3,5-triazine (TPTZ) were all purchased from Tokyo Chemical Industry (Tokyo, Japan) with purity greater than 95%. Stock solutions were freshly prepared in methanol at a final concentration of 5.0 mmol L^−1^ before the measurements. BSA stock solution (0.5 mmol L^−1^) was prepared by dissolving the solid with double deionized water and stored at −20 °C. All the other reagents were analytic grade and double deionized water (18.25 MΩ·cm, 25 °C) from Millipore MilliQ system (Millipore Corporation, Milford, MA, USA) was used.

### 3.2. Apparatus

The cyclic voltammograms (CV) and chronoamperometric experiments (CRAC) were performed with a CHI 660E electrochemical workstation (Shanghai Chenhua Instrumental Corp, Shanghai, China) connected to computer used the software of chi 660e. The DPPH assay and ferric reducing antioxidant power (FRAP) assay were carried out using an ELISA reader (Synergy HT, BioTek, USA).

### 3.3. Spectrophotometric Assays

The DPPH assay was conducted according to a formerly reported 96-well microplate method with some modifications [24]. Water was used as a blank. Briefly, 50 μL sample solutions were firstly incubated with 50 μL BSA or water for 10 min in a 96-well microplate, then 100 μL DPPH reagent (0.2 mmol L^-1^) was added and the microplate was placed in the dark at room temperature for 30 min. After that, the absorbance of the final solutions at 517 nm was recorded on a spectrophotometric microplate reader. The percentage radical scavenging activity values were calculated and the half maximal radical-scavenging activity (IC_50_) values were obtained via graphing method [25]. The antioxidant activities of free flavonoids and those binding with BSA were compared according to their IC_50_ values, and lower value of IC_50_ indicated higher antioxidant activity. Each sample was tested in triplicate.

The FRAP assay was done according to our previous study with a little modifications [12]. Before test, the fresh working solution was prepared by mixing 10 mL acetate buffers (pH 3.6), 1 mL TPTZ solution (in 40 mmol L^−1^ HCl), and 1 mL FeCl_3_·6H_2_O solution (20 mmol L^−1^). The flavonoids or incubated with BSA were respectively allowed to react with the FRAP solution. The absorbance of samples at 595 nm was monitored from 0 min to 15 min with an interval of 1 min.

### 3.4. Electrochemical Assays

A three-electrode system was used including a working electrode (glassy carbon), a reference electrode (Ag|AgCl|3 mol L^−1^ KCl) and a platinum wire as a counter-electrode for CV measurements. The working electrode was cleaned prior to each analysis. The CV was performed by cycling between −0.1 and 1.1 V (versus SCE) at 100 mV/s until a stable voltammograms was obtained. 50 μmol L^−1^ flavonoids in phosphate buffer (pH 7.4) was used to generate higher resolution voltammograms. And the final concentration of BSA solution is 5 μmol L^−1^ to keep the proportion of 10:1 (flavonoid: BSA).

The CRAC assay was performed according to Ferreira’s work with some modifications [17]. CRAC is an electrochemical assay that uses chronoamperometry to directly quantify the antioxidant capacity of samples, and acidic cerium (IV, Ce^4+^) sulfate was used as the oxidant. The decrease of initial concentration of Ce^4+ ^after reacting with the samples was monitored and correlated with the antioxidant capacity of samples using Cottrell equation. Briefly, to carry out the chronoamperometric assays of the flavonoids, 10 mL of CRAC regent were initially deoxygenated with N_2_ for 10 min, and then, a certain volume of flavonoid stock solutions was added keeping the N_2_ stirring for an additional 4 min. A standard curve was obtained from the Ce^4+^ oxidizing solution by varying the Ce^4+^ concentration (a concentration range from 0 to 1.0 mmol L^−1^) against the Cottrell slope (*b*) produced in subsequent chronoamperometric assays. In each assay, the oxidizing solution underwent a deoxygenation process for 10 min prior to the chronoamperometric measurements. Similar to CV, the final concentration of flavonoid stock solution was 25 μmol L^−1^ and the final concentration of BSA solution was 2.5 μmol L^−1^.

## 4. Conclusions

Electrochemical methods including CV and CRAC assay were employed to study the influence of protein - flavonoid interaction on the antioxidant capacity. CV patterns show that almost all the flavonoids show a decreased oxidation peak height in different degrees, which means binding to BSA minimized the chances of flavonoids to be oxidized, and BSA has a certain masking effect for flavonoids. The antioxidant activities of flavonoids were detected by the CRAC method and the results were also compared with the results from the spectrophotometric methods including DPPH and FRAP assays. All the flavonoids showed a decreasing in the antioxidant activity detected by CRAC assay, indicting a “masking effect” of BSA-flavonoid interaction. However, the results from DPPH and FRAP assays were conflicting, which may be attributed to the influence of solvent systems. The present study confirmed the masking effects of BSA on the antioxidant activities of flavonoids, and the masking effects to antioxidant activities were higher as the affinities of BSA for flavonoids increased. Because of the convenience, efficiency and similarity to the physiological environment of the detection system, the CRAC assay shows more potential uses in antioxidant activities evaluations at complicated circumstances.

## Figures and Tables

**Table 1:** Cottrell slopes, ceric reducing/antioxidant capacity (CRAC) values and Trolox equivalent (TE) values for the addition of 25 μmol L^−1^ of antioxidant before and after complexing with BSA.

Antioxidant	*b *(μA s^1/2^)	*CRAC_value _*× 10^6^ ([Ce^3+^]/mol L^−1^)	*TE*
Quercetin	3.34 ± 0.11	294.76 ± 24.02	2.01
BSA-quercetin	0.65 ± 0.08	126.54 ± 17.47	0.86
Morin	1.49 ± 0.18	698.69 ± 39.30	4.78
BSA-morin	0.66 ± 0.08	124.35 ± 17.47	0.85
Fisetin	3.11 ± 0.38	344.98 ± 83.00	2.36
BSA-fisetin	0.43 ± 0.06	174.57 ± 13.10	1.19
Myricetin	3.19 ± 0.09	327.51 ± 19.65	2.24
BSA-myricetin	0.78 ± 0.10	98.15 ± 22.49	0.67
Kaempferol	1.28 ± 0.16	744.51 ± 34.93	5.09
BSA-kaempferol	0.41 ± 0.05	178.94 ± 10.91	1.22
Galangin	2.23 ± 0.27	537.12 ± 58.95	3.67
BSA-galangin	0.43 ± 0.05	174.57 ± 22.49	1.19
Catechin	3.47 ± 0.05	266.38 ± 10.92	1.82
BSA-catechin	0.83 ± 0.10	88.23 ± 10.92	0.60
Trolox	4.02 ± 0.05	146.29 ± 10.91	1.00

**Table 2:** IC_50_ values of 2,2-diphenyl-1-picrylhydrazyl (DPPH), ferric reducing antioxidant power (FRAP) values and corresponding TE values for different flavonoids and BSA-flavonoids with the DPPH assay and FRAP assay.

	DPPH IC_50_ (μmol L^−1^)	TE_DPPH_	FRAP Value (μmol L^−1^ FeSO_4_·7H_2_O)	TE_FRAP_
Quercetin	9.61 ± 0.86	4.58	131.24 ± 4.50	1.20
BSA-quercetin	9.16 ± 0.46	4.81	123.57 ± 10.43	1.13
Morin			128.04 ± 5.37	1.17
BSA-morin			152.74 ± 8.74	1.39
Fisetin	10.33 ± 0.52	4.26	130.01 ± 3.13	1.19
BSA-fisetin	9.26 ± 0.44	4.75	121.45 ± 26.36	1.11
Myricetin	10.48 ± 0.44	4.20	123.60 ± 4.73	1.13
BSA-myricetin	12.45 ± 0.55	3.54	127.58 ± 4.85	1.16
Kaempferol	33.54 ± 8.79	1.31	90.77 ± 3.43	0.82
BSA-kaempferol	40.77 ± 2.20	1.08	119.71 ± 8.32	1.09
Galangin			39.64 ± 3.26	0.36
BSA-galangin			53.87 ± 10.25	0.49
Catechin	29.30 ± 6.34	1.50	91.98 ± 3.00	0.84
BSA-catechin	12.50 ± 0.44	3.52	122.31 ± 5.86	1.12
Trolox	44.02 ± 3.63	1.00	109.61 ± 2.91	1

**Table 3:** TE value changes in proportion of different assays of antioxidant before and after complexation with BSA.

	ΔT%_CRAC_	ΔT%_DPPH_	ΔT%_FRAP_
Quercetin	−57.2	5.0	−5.8
Morin	−82.2	-	18.8
Fisetin	−52.5	11.5	−6.7
Myricetin	−70.1	−15.7	2.7
Kaempferol	−76.0	−17.6	32.9
Galangin	−67.6	-	36.1
Catechin	−67.0	134.7	33.3
